# Efficacy and Safety of New Oral Anticoagulants versus Warfarin in the Resolution of Atrial Fibrillation with Left Atrial/Left Atrial Appendage Thrombus: A Systematic Review and Meta-Analysis

**DOI:** 10.31083/RCM26055

**Published:** 2025-01-20

**Authors:** Guan-lian Mo, Jing Wen, Yu-yu Ye, Yong-qi Lu, Tian-ming Gan, Ying-jie Yang, Jin-yi Li

**Affiliations:** ^1^Department of Cardiology, Affiliated Hospital of Guilin Medical University, 541001 Guilin, Guangxi, China

**Keywords:** new oral anticoagulants, vitamin K antagonist, atrial fibrillation, thrombosis, meta-analysis

## Abstract

**Background:**

To compare the efficacy and safety of novel oral anticoagulants (NOACs) and vitamin K antagonists (VKAs) in nonvalvular atrial fibrillation (NVAF) patients with left atrial/left atrial thrombosis through a systematic review and meta-analysis.

**Methods:**

The CBM (China Biology Medicine disc), CNKI (China National Knowledge Infrastructure), VIP (Chinese Technology Periodical Database), Wanfang, PubMed, Embase, Cochrane Library, and Web of Science databases were searched for relevant studies from their inception to June 30, 2022.

**Results:**

Twelve articles (eight cohort studies and four randomized controlled trials) involving 982 patients were included. Meta-analysis showed that NOACs had a significantly higher thrombolysis rate than VKAs (78.0% vs. 63.5%, odds ratio (OR) = 2.32, 95% confidence interval (CI) 1.71 to 3.15, *p* < 0.0001). Subgroup analysis revealed rivaroxaban to be more effective than VKAs, whereas there was no significant difference between dabigatran and apixaban. There were no significant differences in embolic events, bleeding, or all-cause mortality. Thrombus resolution analysis showed higher left ventricular end-diastolic diameter and smaller left atrial diameter in the effective group than in the ineffective group.

**Conclusions:**

NOACs are more effective in thrombolysis than VKAs in NVAF patients with left atrial thrombosis, and there is no increased risk of adverse events compared with VKAs.

## 1. Introduction

Nonvalvular atrial fibrillation (NVAF) is an independent risk factor for ischemic stroke and leads to a fivefold increase in mortality compared with normal subjects [1]. NVAF is one of the important causes of stroke and cardiovascular events worldwide. According to statistics, the risk of stroke in atrial fibrillation (AF) patients is five times higher than that in the general population, and the incidence of NVAF and associated stroke risk are significantly increased, especially in elderly patients. Pathophysiological mechanisms of NVAF involve atrial electrophysiological changes like structural remodeling, fibrosis, and electrical conduction abnormalities, leading to ineffective atrial contraction and arrhythmia. Structural changes in the atrium, inflammatory responses, and autonomic nervous system effects also contribute to the development and maintenance of NVAF, with underlying diseases and chronic inflammation playing key roles in promoting the arrhythmia [2].

Due to ineffective and irregular rapid atrial contraction and diastolic dysfunction, blood circulation through the left atrium or left atrial appendage can lead to stagnation, resulting in the formation of left atrial thrombus (LAT) or left atrial appendage thrombus (LAAT) [3]. In the evaluation of biomarkers for left atrial appendage thrombosis, MPV (mean platelet volume), NT-proBNP (N-terminal pro-B type natriuretic peptide), RDW (red blood cell distribution width) and CHA2DS2-VASc score have shown certain potential. In-depth study of these biomarkers can help reveal their true value in clinical applications and inform future therapeutic strategies [4]. LAT or LAAT serves as the source of embolus in AF [5]. Therefore, thrombus resolution is crucial in preventing embolic events in AF patients with LAT or LAAT.

Anticoagulant drugs in the clinical application for AF can be categorized into two groups, namely, warfarin and vitamin K antagonists (VKAs) and non-vitamin K antagonist oral anticoagulants (NOACs) such as dabigatran, rivaroxaban, and apixaban. Before the introduction of NOACs, studies had demonstrated that warfarin reduced the risk of stroke by 66% [6]. However, patients with AF often struggle with compliance and coordination due to the influence of food and drug interactions, the need for the frequent monitoring of coagulation, and a narrow treatment time window associated with warfarin use. Recent studies have confirmed that NOACs are more effective than warfarin in preventing stroke and reducing bleeding events in patients with NVAF [7, 8, 9]. Consequently, NOACs are now recommended in the guidelines for the prevention of embolic events in patients with NVAF [10].

In AF patients with LAT or LAAT, the primary challenge is dissolving the thrombus. Karwowski *et al*. [11] have confirmed that a significant proportion (approximately 58%) of the population is affected by LAT/LAAT. Although case reports [12, 13] and clinical research [14, 15, 16] have shown that NOACs are as effective and safe as VKAs in treating AF patients with LAT/LAAT, there are limited data on the efficacy and safety of LAT/LAAT resolution with NOACs. The optimal regimen for thrombolytic therapy in AF patients with LAT/LAAT remains unclear. Therefore, this study aimed to systematically evaluate the efficacy and safety of NOACs and VKAs in treating LAT/LAAT through meta-analysis. The results of this study could provide valuable insights for the clinical management of AF patients with LAT/LAAT.

## 2. Data Sources

We searched both Chinese (CBM (China Biology Medicine disc), CNKI (China National Knowledge Infrastructure), VIP (Chinese Technology Periodical Database), and Wanfang) and English (PubMed, Embase, Cochrane Library, and Web of Science) databases without imposing any restrictions on the study type. The databases were searched from their inception to June 30, 2022. A manual reference search was conducted to meet inclusion criteria and enhance the reliability of the study conclusions. A search formula using keywords was structured as follows: (“new oral anticoagulant” OR “non-vitamin K oral anticoagulants” OR rivaroxaban OR dabigatran OR apixaban OR edoxaban) AND (“atrial fibrillation” OR “left atrium” OR “left atrial appendage” OR thrombus) AND (“resolution” OR “dissolution” OR “decomposition” OR “dissolve” OR “thrombolysis”) AND (“randomised controlled trial” OR “controlled clinical trial” OR randomised OR randomly OR trial). The search strategy used in this study is outlined in Table 1. The reporting of the study followed the Preferred Reporting Items for Systematic Reviews and Meta-Analyses (PRISMA).

**Table 1.** **Search strategy used in this study**.

No	Search terms
1	new oral anticoagulant .ti,mesh.
2	non-vitamin K oral anticoagulants .ti,ab.
3	atrial fibrillation .ti,ab .
4	or 1–3
5	rivaroxaban.ti,ab.
6	dabigatran.ti,ab.
7	apixaban .ti,ab.
8	edoxaban .ti,ab.
9	or 5–8
10	resolution
11	resolution
12	decomposition
13	dissolve
14	or 10–13
15	left atrium .ti,ab.
16	left atrial appendage .ti,ab.
17	or 15–16
18	thrombus .ti,ab.
19	thrombolysis .ti,ab.
20	or 18–19
21	randomised controlled trial.pt.
22	controlled clinical trial.pt.
23	randomised.ab.
24	randomly.ab.
25	trial.ab.
26	or 21–25
27	exp animals/not humans.sh.
28	26 not 27
29	4 and 9 and 14 and 17 and 20 and 26 and 28

### 2.1 Eligibility Criteria

The inclusion criteria for this study were patients with NVAF and LAT/LAAT as diagnosed by transesophageal echocardiography (TEE) or multirow helical computed tomography (MDCT). Left atrial appendage sludge (LAAS) was also included due to high thrombosis risk [17]. In terms of interventions, the test group received NOACs (rivaroxaban, apixaban, xaban, and dabigatran) without dose limitation and the control group received oral VKA. Regarding outcome measures, efficacy indicators included LAT/LAAT resolution; safety indicators included embolic events, bleeding events, cardiovascular adverse events, and all-cause deaths.

The exclusion criteria were as follows: ① studies with patients with valvular AF; ② studies using interfering drugs (e.g., heparin, aspirin, and clopidogrel); ③ studies with less than 20 cases (the exclusion of studies with a sample size of fewer than 20 cases in a meta-analysis is a methodological strategy employed to enhance the quality, reliability, and validity of the pooled findings by minimizing biases, ensuring statistical power, improving generalizability, and enhancing the precision and stability of effect estimates.); ④ studies with unknown outcomes, an abstract-only format, or no access to the full text or data; ⑤ case reports, reviews, conference summaries, animal experiments, meta-analyses, or “other” article types; or ⑥ duplicate articles or articles from the same institution (in such cases, we selected the one with the longest follow-up and the largest sample size).

### 2.2 Selection of Studies, Data Extraction, and Management

Two researchers conducted the literature search and data extraction independently. Disagreements were resolved through consultation with a third researcher. The following data were extracted: first author, publication year, study type, gender ratio, age, AF type, follow-up duration, follow-up protocol (TEE/MDCT), NOAC dose, sample size, outcome measures (LAT/LAAT resolution, embolic events, bleeding events, cardiovascular events, and all-cause death), and potential factors affecting thrombus resolution (AF subtype, left atrial diameter (LAD), and left ventricular end-diastolic diameter (LVEDd)).

### 2.3 Certainty of Evidence

The included articles comprised observational studies (prospective and retrospective cohort studies) and randomized controlled trials (RCTs). The quality of cohort studies was assessed using the Newcastle–Ottawa Scale (NOS), while RCTs were evaluated using the Cochrane Risk of Bias assessment tool. Two researchers independently conducted the evaluation, with a third researcher resolving any disagreements.

### 2.4 Data Synthesis and Analysis

This study used Revman 5.3 software (Cochrane Collaboration, Oxford, United Kingdom) for comprehensive quantitative analysis. Heterogeneity Test: The study utilized Q-value statistic tests and I^2^ tests to assess heterogeneity among studies. A funnel plot was also employed for a preliminary evaluation of heterogeneity. Studies were considered homogeneous and within an acceptable range if *p* > 0.10 or I^2^ ≤50%, whereas further investigation was warranted for *p* < 0.10 or I^2^ ≥50%, potentially requiring error checks, subgroup analysis, and sensitivity analysis, particularly for substantial heterogeneity (I^2^ ≥75%). Effect Model: Depending on heterogeneity levels, either the Fixed effect model or Random effect model was utilized for effect size pooling using the Mantel-Haenszel (M-H) method. Small heterogeneity led to the Fixed effect model application, while large heterogeneity required the Random effect model, with division into two groups to address clinical heterogeneity and ensure accurate effect size estimation. Effect measures: Odds ratio (OR) and 95% confidence interval (CI) were employed for binary variables, with interpretation based on the position in the forest plot. Mean difference (MD) and 95% CI were used for continuous variables, with statistical significance (*p* < 0.05) indicating a significant difference. The OR and MD values guided the impact assessment of intervention measures on observed outcomes. Sensitivity Analysis: Various techniques, such as modifying conditions, clipping and filling, and individually excluding studies, were employed to gauge model reliability and assess sources of heterogeneity. One-at-a-time exclusion of individual studies followed by repeat meta-analysis determined the influence on overall effect size, highlighting key studies impacting outcomes significantly. Bias Evaluation: The study used a funnel plot to discern publication bias, with an ideal pattern reflecting a symmetrical distribution of large and small sample studies. Detection of missing negative results in the plot indicated potential publication bias.

## 3. Results

### 3.1 Characteristics of the Included Studies

**Fig. 1.**
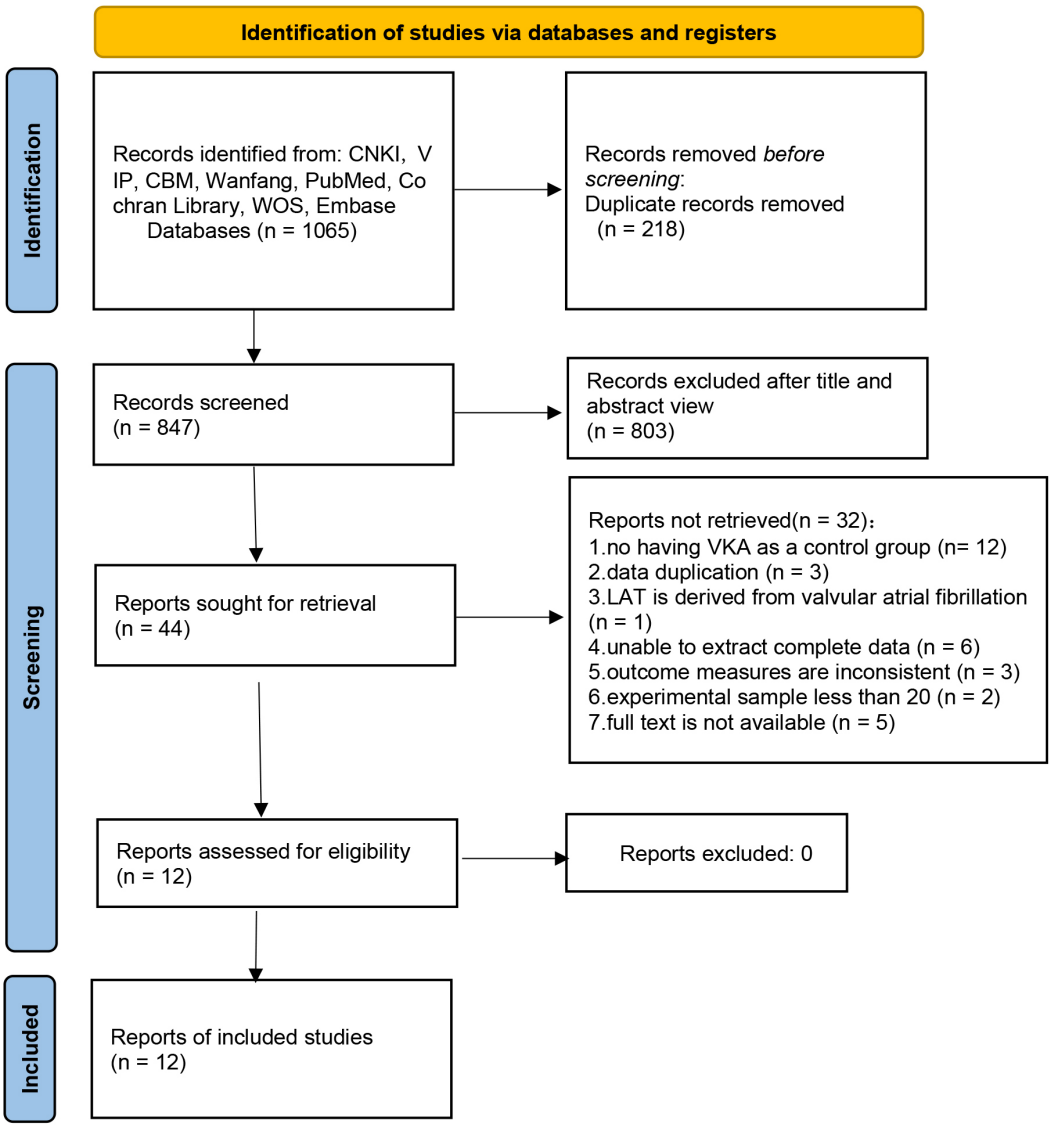
**Flowchart of literature screening**. Note: CNKI, China National Knowledge Infrastructure; VIP, Chinese Technology Periodical Database; CBM, China Biology Medicine disc; WOS, Web of Science; LAT, left atrial thrombus.

The systematic search of databases retrieved 1065 articles. After removing duplicates, 847 articles remained. Following the exclusion of medical records, reviews, conference abstracts, animal experiments, meta-analyses, guidelines, and unrelated articles, 44 articles remained. Full-text evaluation resulted in the exclusion of articles with no VKA control; articles with repeated data, valvular AF, incomplete data, mismatched outcome indicators, and inadequate sample size; and articles without access to full text, leaving 12 articles for inclusion. The literature screening process is illustrated in Fig. 1.

The 12 included articles comprised eight cohort studies (four prospective [18, 19, 20, 21] and four retrospective [22, 23, 24, 25]) and four RCTs [26, 27, 28, 29]. Our study included 982 NVAF patients with LAT/LAAT, with 490 in the experimental group receiving NOACs and 492 in the control group receiving VKAs. The baseline characteristics are shown in Table 2 (Ref. [18, 19, 20, 21, 22, 23, 24, 25, 26, 27, 28, 29]).

**Table 2.** **Basic information included in the study**.

Study	Source	Study type	Gender (male%)	Age (year)	PeAF (n %)	Thrombus	Intervention study	Sample capacity		fl.up time
								NOACs	VKAs	
Huang *et al*. [26], 2018	China	RCT	53.7	75–80 y	100.0	LAT	Rivaroxaban 15 mg QD	18	36	3 m
						LAAT	warfarin (high, low intensity)			6 m
										12 m
He *et al*. [28], 2019	China	RCT	55.0	72.84 ± 4.29 y/72.70 ± 4.25 y	75.0	LAT	Rivaroxaban 20 mg QD	50	50	2 m
							warfarin			4 m
										6 m
Xue *et al*. [27], 2020	China	RCT	64.9	67.53 ± 9.48 y/65.31 ± 8.67 y	unclear	LAT	Rivaroxaban 20 mg QD	74	74	6 m
							warfarin			
Ke, *et al*. [29], 2019	China	RCT	82.5	64.20 ± 10.50 y/63.70 ± 86.0 y	unclear	LAT	Rivaroxaban 20 mg QD	40	40	6 w
						LAAT	warfarin			3 m
Yan *et al*. [18], 2018	China	cohort study	77.4	60.3 ± 10.6 y	74.3	LAT	Rivaroxaban 20 mg QD	64	31	3 w
						LAAS	Dabigatran 110 mg BID			
							warfarin			
Wu *et al*. [23], 2018	Britain	cohort study	56.8	63 y (median)	61.4	LAT	Apixaban, dabigatran, rivaroxaban, warfarin (dose unclear)	20	24	4.2 m (median)
Hao *et al*. [19], 2015	China	cohort study	87.8	57.7 ± 7.4 y	41.5	LAT	Dabigatran at 150 mg BID	19	22	3 m
						several RAT	warfarin			
Hussain *et al*. [22], 2019	America	cohort study	68.9	63.2 ± 15.3 y	40.0	LAAT	Apixaban, dabigatran, rivaroxaban, and warfarin (Dose of unclear)	22	23	3 w–1 y
						LAAS				
Yang *et al*. [20], 2019	China	cohort study	72.2	63.5 ± 10.9 y	76.4	LAT	Rivaroxaban 15/20 mg QD, dabigatran 110/150 mg BID, and warfarin	70	17	101.5 d
						LAAT				
Mazur *et al*. [25], 2021	Russia	cohort study	76.5	59.7 ± 9.8 y	100.0	LAAT	Rivaroxaban 20 mg QD	31	37	33 ± 14.2 d
							Dabigatran 150 mg BID apixaban 5 mg BID			
							warfarin			
Nelles *et al*. [21], 2021	Germany	cohort study	57.7	76 ± 8 y	43.6	LAAT	Apixaban, dabigatran, Rivaroxaban, Eaban, VKAs (dose unclear)	53	29	About 1 y
Faggiano *et al*. [24], 2022	Italy	cohort study	unclear	71 y (median)	unclear	LAT	unclear	50	107	39 d
						LAAT				(median)

Note: RCT, randomized controlled trial; PeAF, persistent atrial fibrillation; LAT, left atrial thrombus; LAAT, left atrial appendage thrombus; LAAS, left atrial appendage sludge; RAT, right atrial thrombus; m, month; w, week; y, year; d, day; BID, bis in die; QD, quaque die; NOACs, novel oral anticoagulants; VKAs, vitamin K antagonists.

### 3.2 Quality Evaluation and Risk of Bias

The Cochrane Risk of Bias Tool and the NOS were used for bias assessment. Cochrane Risk of Bias Tool: ① Selection Bias: This domain assesses the random sequence generation and allocation concealment methods used in the study. ② Performance Bias: Evaluates blinding of participants and personnel to prevent biases in study conduct. ③ Detection Bias: Determines if outcome assessors were blinded to prevent subjective judgments. ④ Attrition Bias: Examines missing data and dropout rates to assess the impact on study outcomes. ⑤ Reporting Bias: Assesses selective outcome reporting that can lead to bias in the interpretation of results. ⑥ Other Bias: Considers any additional biases not covered in the other domains.

NOS: ① Selection: Evaluates the representativeness of the study groups and the selection of controls. ② Comparability: Considers the comparability of cases and controls based on study design and adjustment for confounders. ③ Exposure/Outcome: Assesses the ascertainment of exposure for cases and controls, and the demonstration of outcomes.

Handling Studies with High Risk of Bias: If a study is identified to have a high risk of bias based on the Cochrane Risk of Bias Tool or NOS, one approach is to perform a sensitivity analysis to evaluate the impact of excluding these studies on the overall results. In sensitivity analysis, the meta-analysis is conducted with and without the high-risk studies to assess the robustness of the findings. Interpretation of results should consider the potential influence of studies with a high risk of bias on the pooled effect estimates. If sensitivity analysis indicates a significant impact on results or the conclusions drawn, alternative strategies, such as subgroup analysis or additional assessments of study quality, may be necessary.

**Fig. 2.**
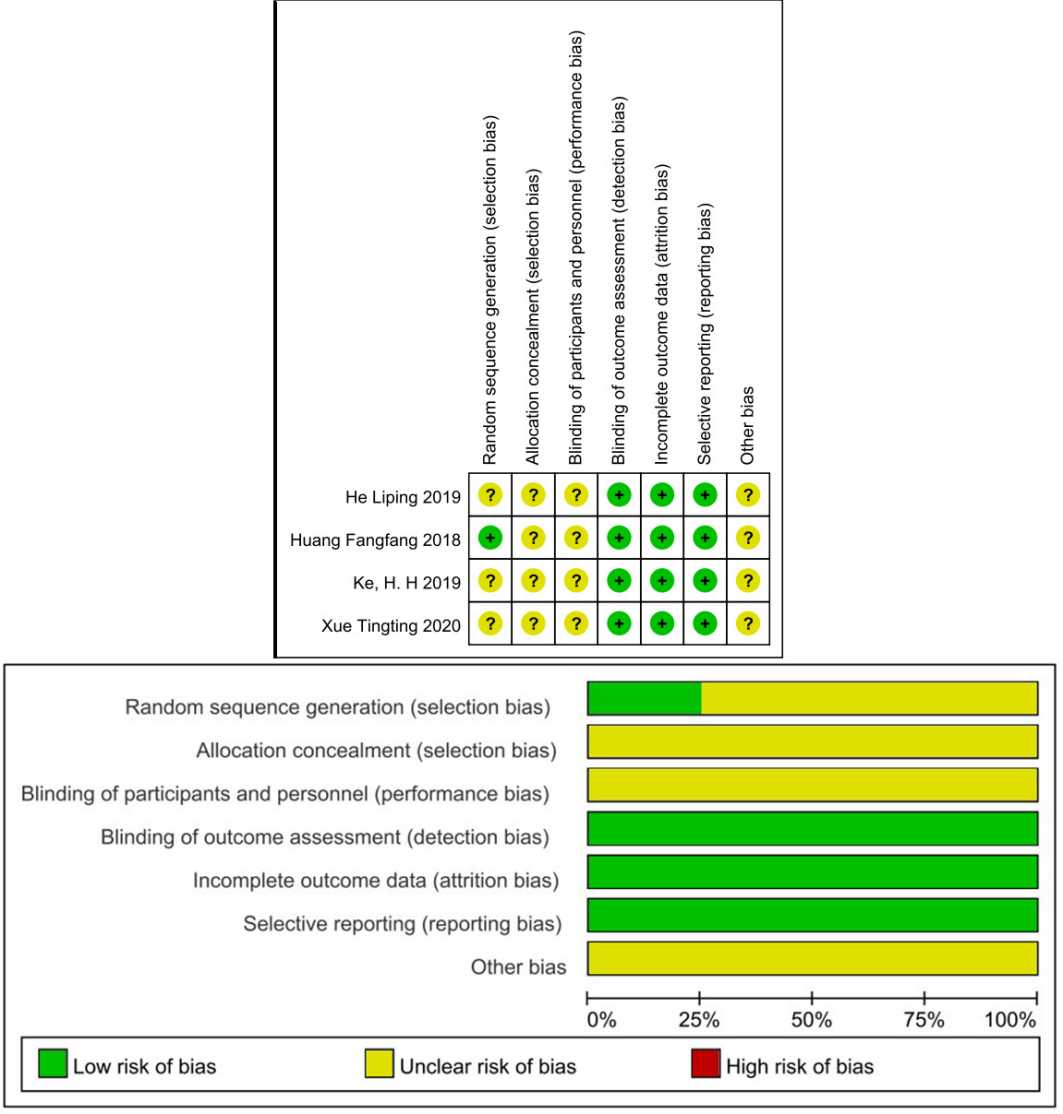
**Risk of bias**.

Table 3 shows the NOS scores of the included cohort studies. Namely, two studies scored 8 stars [19, 22], four studies scored 7 stars [20, 21, 23, 24], one study scored 6 stars [18], and one study scored 5 stars [30]. The Cochrane Risk of Bias assessment tool was used for the included RCTs, as depicted in Fig. 2. Allocation and selection bias had uncertain risk due to the lack of information in most articles. Blinding was not described, resulting in uncertain risk. Follow-up and reporting bias had low risk. None of the articles exhibited selective reporting bias.

**Table 3.** **Newcastle–Ottawa Scale (NOS) scores of the included cohort studies**.

Bring into study	Publish a particular year	Selection	Comparability	Outcome	Total points
Yan *et al*. [18]	2018	★ ★ ★	★	★ ★	Six stars
Wu *et al*. [23]	2018	★ ★ ★	★	★ ★ ★	Seven stars
Hao *et al*. [19]	2015	★ ★ ★	★ ★	★ ★ ★	Eight stars
Hussain *et al*. [22]	2019	★ ★ ★	★ ★	★ ★ ★	Eight stars
Yang *et al*. [20]	2019	★ ★ ★	★	★ ★ ★	Seven stars
Mazur *et al*. [25]	2021	★ ★	★	★ ★	Five stars
Nelles *et al*. [21]	2021	★ ★ ★	★ ★	★ ★	Seven stars
Faggiano *et al*. [24]	2022	★ ★ ★	★ ★	★ ★	Seven stars

Note: The symbol ★ represents NOS (Newcastle-Ottawa Scale) scores, which is used to assess the quality of observational studies, including cohort studies.

### 3.3 Outcome Measures

#### 3.3.1 Efficacy of NOACs in the Resolution of NVAF Patients with LAAT

3.3.1.1 NOACs vs. VKAs

**Fig. 3.**
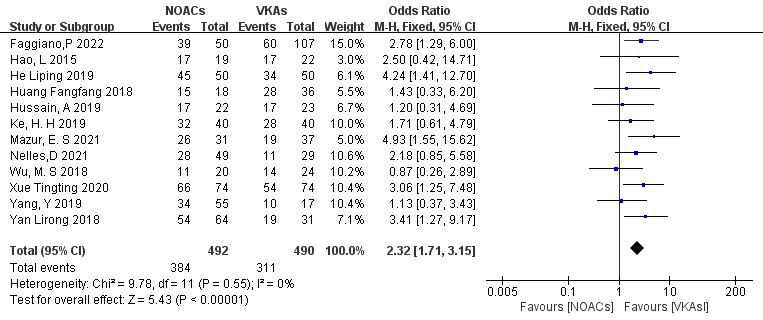
**Comparison of the thrombolysis efficacy between novel oral anticoagulants (NOACs) and vitamin K antagonists (VKAs)**. CI, confidence interval; M-H, Mantel-Haenszel.

All 12 included articles described the comparison of NOACs and VKAs in 982 patients [18, 19, 20, 21, 22, 23, 24, 25, 26, 27, 28, 29]. Among 492 patients treated with NOACs and 490 patients treated with VKAs, there was no heterogeneity (*p* = 0.55, I^2^ = 0%), allowing for a fixed-effects model analysis. The results indicated a higher thrombolysis rate with NOACs than with VKAs (78.0% vs. 63.5%), with a statistically significant difference (OR = 2.32, 95% CI 1.71 to 3.15, *p* < 0.0001) (Fig. 3).

3.3.1.2 Rivaroxaban vs. VKAs

**Fig. 4.**
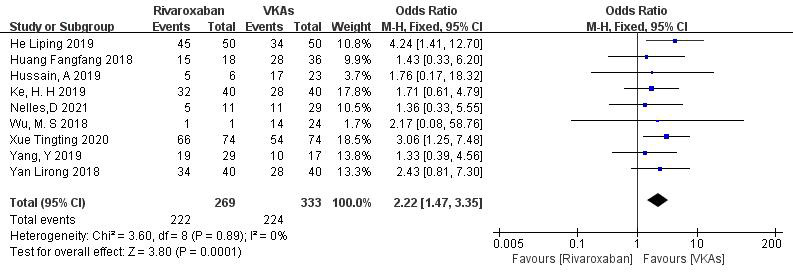
**Comparison of thrombolytic efficacy between rivaroxaban and vitamin K antagonist**. CI, confidence interval; VKAs, vitamin K antagonists; M-H, Mantel-Haenszel.

Nine studies (totaling 602 patients) comparing thrombolysis rates of rivaroxaban and VKAs were included [18, 20, 21, 22, 23, 26, 27, 28, 29]. Among 269 patients receiving rivaroxaban and 333 patients receiving VKAs, there was no significant heterogeneity (*p* = 0.89, I^2^ = 0%). Therefore, a fixed-effects model was used for meta-analysis. The results showed a higher thrombolysis rate with rivaroxaban than with VKAs (82.5% vs. 67.3%), with a statistically significant difference (OR = 2.22, 95% CI 1.47 to 3.35, *p* = 0.0001) (Fig. 4).

3.3.1.3 Dabigatran vs. VKAs

**Fig. 5.**
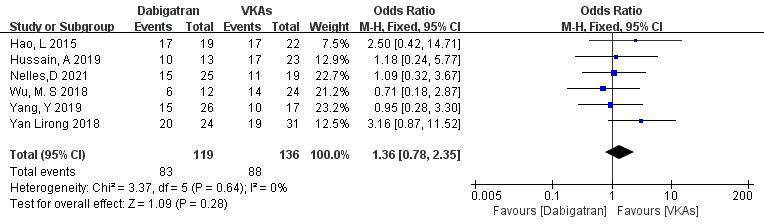
**Comparison of thrombolysis efficacy between dabigatran and vitamin K antagonist**. CI, confidence interval; VKAs, vitamin K antagonists; M-H, Mantel-Haenszel.

Six studies (totaling 255 patients) comparing thrombolysis rates between dabigatran and VKAs were included [18, 19, 20, 21, 22, 23]. Among 119 patients receiving dabigatran and 136 patients receiving VKAs, there was no significant heterogeneity (*p* = 0.64, I^2^ = 0%). Therefore, a fixed-effects model was used for meta-analysis. The results showed no significant difference in thrombolysis rates between dabigatran and VKAs (69.7% vs. 64.7%; OR = 1.36, 95% CI 0.78 to 2.35, *p* = 0.28) (Fig. 5).

3.3.1.4 Apixaban vs. VKAs

**Fig. 6.**
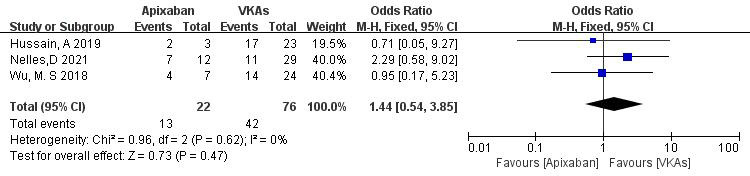
**Comparison of thrombolysis efficacy between apixaban and vitamin K antagonist**. CI, confidence interval; VKAs, vitamin K antagonists; M-H, Mantel-Haenszel.

Three studies (totaling 98 patients) comparing thrombolysis rates between apixaban and VKAs were included [21, 22, 23]. Among 22 patients receiving apixaban and 76 patients receiving VKAs, there was no significant heterogeneity (*p* = 0.62, I^2^ = 0%). Therefore, a fixed-effects model was used for meta-analysis. The results showed no significant difference in thrombolysis rates between apixaban and VKAs (59.1% vs. 55.3%; OR = 1.44, 95% CI 0.54 to 3.85, *p* = 0.47) (Fig. 6).

3.3.1.5 Rivaroxaban vs. Dabigatran

**Fig. 7.**
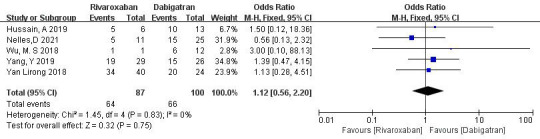
**Comparison of thrombolysis efficacy between rivaroxaban and dabigatran**. CI, confidence interval; M-H, Mantel-Haenszel.

Five studies (totaling 187 patients) comparing thrombolysis rates between rivaroxaban and dabigatran were included [18, 20, 21, 22, 23]. Among 87 patients receiving rivaroxaban and 100 patients receiving dabigatran, there was no significant heterogeneity (*p* = 0.83, I^2^ = 0%). Thus, a fixed-effects model was used for meta-analysis. The results showed no significant difference in thrombolysis rates between rivaroxaban and dabigatran (73.6% vs. 55.3%; OR = 1.12, 95% CI 0.56 to 2.20, *p* = 0.75) (Fig. 7).

#### 3.3.2 Efficacy of Thrombolysis with NOACs and VKAs at Different Follow-Up Times

**Fig. 8.**
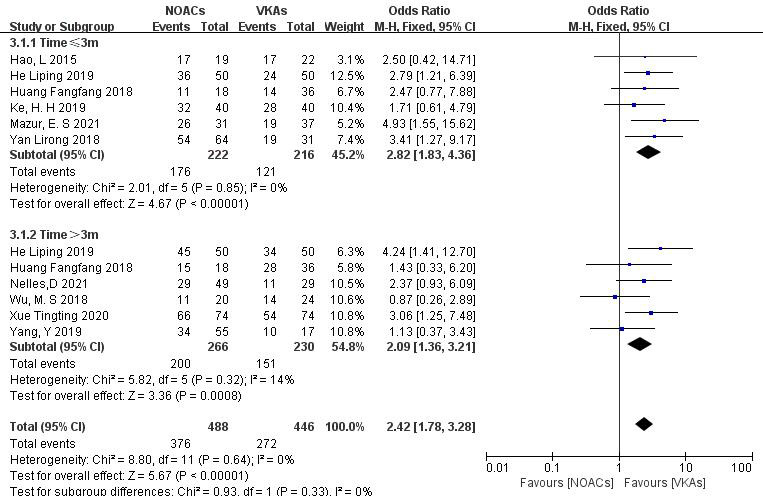
**Comparison of thrombolysis efficacy between NOACs and VKAs at different follow-up times**. CI, confidence interval; VKAs, vitamin K antagonists; NOACs, novel oral anticoagulants; M-H, Mantel-Haenszel.

Subgroup analysis was conducted to compare the effect of follow-up time on thrombolysis rates between NOACs and VKAs. (1) In the studies with a 3-month follow-up (six articles [18, 19, 25, 26, 28, 29], 438 patients), NOACs had a higher thrombolysis rate than VKAs (79.3% vs. 56.2%; OR = 2.82, 95% CI 1.83 to 4.36, *p* < 0.00001). (2) In the studies with >3 months of follow-up (six articles [20, 21, 23, 26, 27, 28], 496 patients), NOACs also had a higher thrombolysis rate than VKAs (77.0% vs. 70.0%; OR = 2.09, 95% CI 1.36 to 3.21, *p* = 0.0008). Follow-up time did not affect the thrombolysis rate, with NOACs consistently demonstrating superior thrombolytic effects (Fig. 8).

#### 3.3.3 Efficacy of Thrombolysis between NOACs and VKAs in Different Types of Studies

**Fig. 9.**
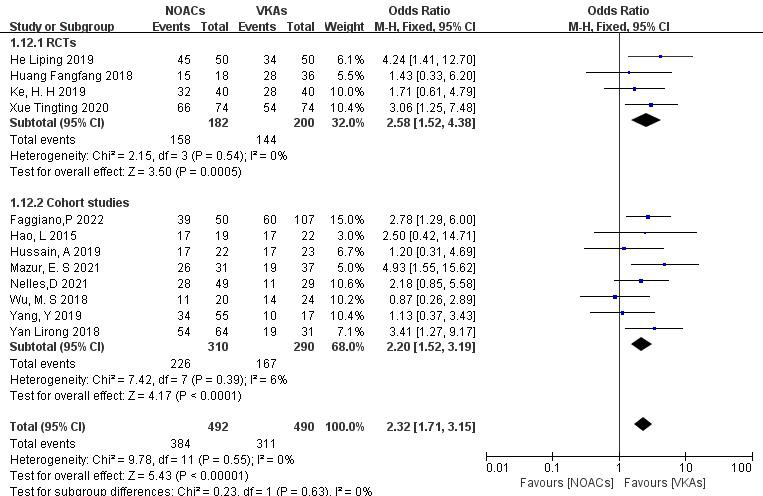
**Efficacy of thrombolysis between NOACs and VKAs in different types of studies**. CI, confidence interval; VKAs, vitamin K antagonists; NOACs, novel oral anticoagulants; M-H, Mantel-Haenszel; RCTs, randomized controlled trials.

To assess the impact of study type, subgroup analysis was conducted on RCTs and cohort studies. (1) In the RCTs (four articles [26, 27, 28, 29], 382 patients), NOACs showed higher thrombosis resolution rates than VKAs (86.8% vs. 72.0%; OR = 2.58, 95% CI 1.52 to 4.38, *p* = 0.0005). (2) In the cohort studies (eight articles [18, 19, 20, 21, 22, 23, 24, 25], 600 patients), NOACs demonstrated higher thrombolysis rates than VKAs (72.9% vs. 57.6%; OR = 2.20, 95% CI 1.52 to 3.19, *p* < 0.0001). The subgroup analysis confirmed the consistent superiority of NOACs in thrombolysis across different types of studies (Fig. 9).

#### 3.3.4 Safety of NOACs in the Resolution of NVAF Patients with LAAT

3.3.4.1 Incidence of Stroke or Embolic Events

**Fig. 10.**
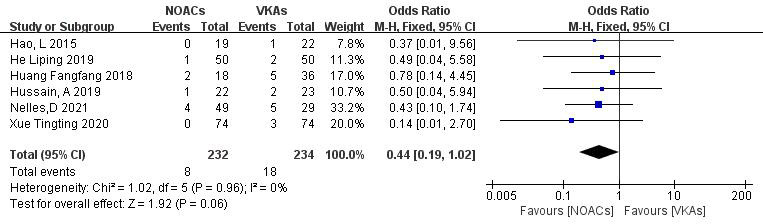
**Comparison of the rate of embolic events between NOACs and VKAs**. CI, confidence interval; VKAs, vitamin K antagonists; NOACs, novel oral anticoagulants; M-H, Mantel-Haenszel.

Six articles [19, 21, 22, 26, 27, 28] compared the incidence of stroke or systemic circulation embolism. Two studies [18, 29] reported no new embolic events in both groups during the follow-up. A total of 466 patients were included, with 232 in the NOAC group and 234 in the VKA group. The heterogeneity test showed no significant heterogeneity (*p* = 0.96, I^2^ = 0%); thus, a fixed-effect model was used. The results demonstrated no significant differences in the incidence of stroke or embolic events between NOACs and VKAs (3.4% vs. 7.7%; OR = 0.44, 95% CI 0.19 to 1.02, *p* = 0.83) (Fig. 10).

3.3.4.2 Incidence of Bleeding Events

**Fig. 11.**
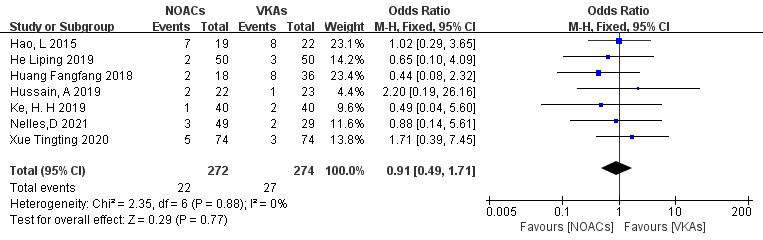
**Comparison of the rate of bleeding events between NOACs and VKAs**. CI, confidence interval; VKAs, vitamin K antagonists; NOACs, novel oral anticoagulants; M-H, Mantel-Haenszel.

Seven studies [19, 21, 22, 26, 27, 28, 29] examined bleeding events, including minor and major bleeding. In the study involving 546 patients, with 272 in the NOAC group and 274 in the VKA group, there were 22 and 27 bleeding events, respectively. There was no significant heterogeneity (*p* = 0.88, I^2^ = 0%), and a fixed-effects model was used. The results indicated no significant difference in bleeding event incidence between NOACs and VKAs (8.1% vs. 9.9%; OR = 0.91, 95% CI 0.49 to 1.71, *p* = 0.77) (Fig. 11).

#### 3.3.5 All-Cause Mortality between NOACs and VKAs

**Fig. 12.**
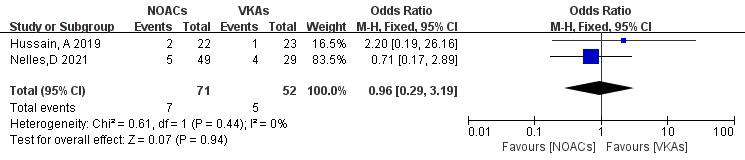
**Death event incidence in comparison of NOACs and VKAs**. CI, confidence interval; VKAs, vitamin K antagonists; NOACs, novel oral anticoagulants; M-H, Mantel-Haenszel.

Only two articles [21, 22] reported mortality rates in NOAC and VKA thrombolysis. In the study, 123 patients were included, with 71 in the NOAC group and 52 in the VKA group; there were seven and five mortality outcomes, respectively. No significant heterogeneity was found (*p* = 0.44, I^2^ = 0%), and a fixed-effects model was used. The results showed no significant difference in death rates between NOACs and VKAs (9.9% vs. 9.6%; OR = 0.96, 95% CI 0.29 to 3.19, *p* = 0.94) (Fig. 12).

#### 3.3.6 Comparison of the Characteristics between the Effective and Ineffective Thrombolysis Groups

3.3.6.1 Comparison of LVEDd, Left Ventricular Ejection Fraction (LVEF), and LAD

**Fig. 13.**
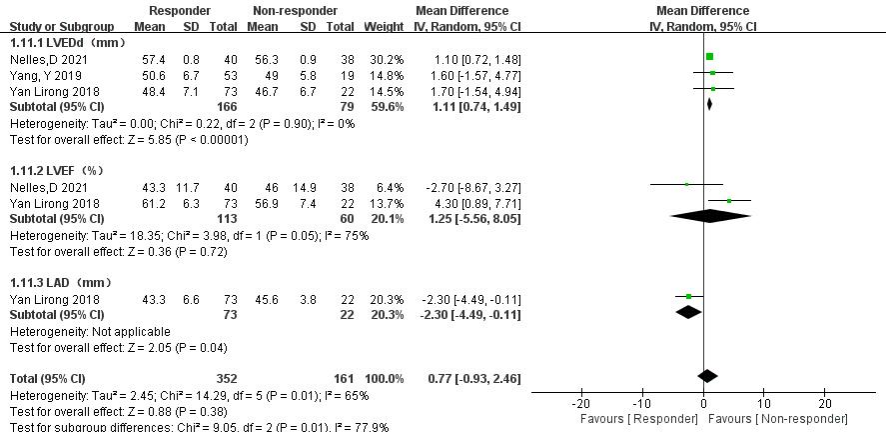
**Left ventricular end-diastolic diameter (LVEDd), left ventricular ejection fraction (LVEF), and left atrial diameter (LAD) between the effective and ineffective group**. CI, confidence interval; SD, standard deviation; IV, instrumental variable.

In three articles [18, 20, 21], data comparing LVEDd, LVEF, and LAD between thrombolysis effectiveness groups were analyzed. Significant heterogeneity was observed in the LVEF group, but further subgroup analysis was not possible due to limited relevant literature. Therefore, a random-effects model was used. The following results were obtained: (1) LVEDd: The ineffective group had a significantly reduced LVEDd compared with the effective group (MD = 1.11, 95% CI 0.74 to 1.49, *p* < 0.00001). (2) LVEF: There was no significant difference in LVEF between the effective and ineffective groups (MD = 1.25, 95% CI –5.56 to 8.05, *p* = 0.72). (3) LAD: The effective group showed a significantly smaller LAD than the ineffective group (MD = –2.30, 95% CI –4.49 to –0.11, *p* = 0.04) (Fig. 13).

3.3.6.2 Comparison of the Subtypes of AF

**Fig. 14.**
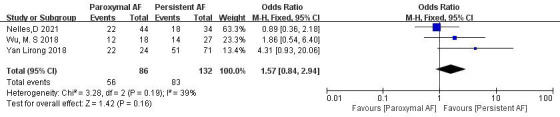
**Comparison of thrombolysis rates for different subtypes of AF**. AF, atrial fibrillation; CI, confidence interval; M-H, Mantel-Haenszel.

Three articles [18, 21, 23] reported AF subtypes. In the study, there were a total of 218 patients. Of 86 patients in the paroxysmal AF group, thrombolysis was achieved in 56, whereas of 132 patients in the persistent AF group, thrombolysis was achieved in 83. Significant heterogeneity was acceptable (*p* = 0.19, I^2^ = 39%), and a fixed-effect model was used. The meta-analysis showed no significant difference in thrombolysis rate between paroxysmal and persistent AF patients (65.1% vs. 62.9%; OR = 1.57, 95% CI 0.84 to 2.94, *p* = 0.16) (Fig. 14).

#### 3.3.7 Sensitivity Analysis

Exclusion of NOACs and VKAs for LAT/LAAT in NVAF patients resulted in similar findings for thrombosis rate, bleeding risk, embolism risk, and mortality compared with the overall meta-analysis. Individual studies had minimal impact on the overall effect size, and the results of each study comparison were stable.

#### 3.3.8 Assessment of Publication Bias

**Fig. 15.**
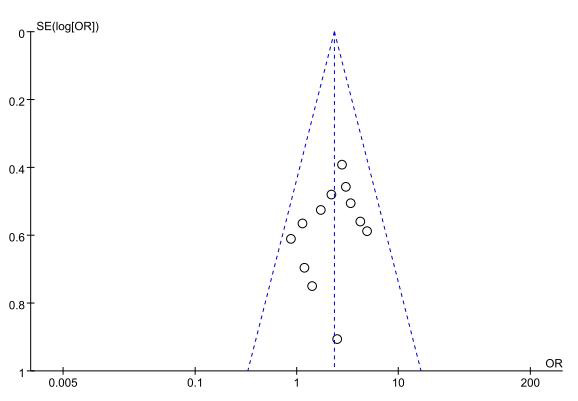
**Funnel plot of thrombolytic efficacy between NOACs and VKAs**. VKAs, vitamin K antagonists; NOACs, novel oral anticoagulants; OR, odds ratio.

The funnel plot results (Fig. 15) demonstrated that the included studies in each outcome were within the 95% CI of the funnel plot. The study points were also distributed symmetrically, indicating no significant publication bias.

## 4. Discussion

We performed this meta-analysis of four RCTs and eight cohort studies with 982 patients to investigate the efficacy of NOACs vs. VKAs in the resolution of AF with LAT/LAAT. The results showed that the thrombolysis rate of NOACs was higher than that of VKAs; NOACs did not increase the risk of stroke or bleeding during the application and had more obvious advantages for thrombosis treatment of NVAF.

The incidence of LAAT in AF patients ranges from 0.5% to 14% [31, 32]. Antithrombotic therapy is crucial in patients with LAT, as it is an absolute contraindication to radiofrequency ablation. There are limited studies on thrombolytic treatment for NVAF with LAT/LAAT, and there have been no large RCTs to guide treatment. This study directly compared the efficacy and safety of NOACs and VKAs for thrombolytic therapy in NVAF with LAT/LAAT. Warfarin, a representative VKA, effectively reduces stroke risk but has limitations in clinical use, such as food and drug interactions, frequent monitoring of international normalized ratio (INR), and individual dose–response variability [8]. Current studies suggest maintaining an INR between 2.0 and 3.0 for effective anticoagulation without increasing bleeding risk [33, 34]. Most studies in the literature have used an INR range of 2.0–3.0 for the warfarin control group. However, the RCT by Huang Fangfang *et al*. [26] divided warfarin into low-intensity (INR 1.5–2.0) and standard-intensity (INR 2.0–3.0) groups, and they found that the effects in the low-intensity group were not superior, but there was a reduced risk of bleeding.

Dabigatran, a direct thrombin inhibitor, has demonstrated a lower prevalence of left heart thrombosis than warfarin in combination therapy [35]. Two common clinical doses of dabigatran (110 mg bis in die (BID) and 150 mg BID) have different efficacy and safety profiles. The 110 mg BID dose is comparable to warfarin in terms of efficacy and reduces bleeding risk, while the 150 mg BID dose increases stroke prevention ability but has comparable bleeding risk to warfarin. Study of Lip *et al*. [36] focused on the thrombolysis effect of dabigatran and included studies using both doses. However, there is limited literature on dose grouping, preventing further verification of different doses’ effects on efficacy and safety. Dabigatran has shown advantages over VKAs in stroke prevention and reducing bleeding risk [8]. Ongoing clinical trials, such as RE-LATED AF-AFNET [35], are studying thrombolysis efficacy of dabigatran compared with warfarin, with results awaited. For patients over 80 years old, the recommended dose is 110 mg twice daily. This article focuses on dabigatran’s thrombolytic effects; however, due to insufficient literature on dosing variations, the impact of different doses on efficacy and safety remains unverified.

Rivaroxaban is a factor Xa inhibitor that effectively inhibits thrombin formation. Compared with warfarin, rivaroxaban has high bioavailability and is not affected by food and drug interactions. The ROCKET-AF [37] study has extensively evaluated the efficacy and safety of rivaroxaban in preventing stroke events. Previous studies, including the X-TRA study [38], have demonstrated the thrombolytic effect of rivaroxaban, making it a viable option for NVAF with LAT. The mechanism of rivaroxaban’s thrombolytic effect involves inhibiting thrombin release from blood clots and increasing fibrinolytic activity [29]. This study includes abundant data on rivaroxaban for LAT in NVAF patients. Subgroup analysis confirmed that rivaroxaban performs better than VKAs in resolving thrombosis in AF patients. Current expert consensus recommends a daily dose of 20 mg taken with food; no adjustment is needed for those over 65 years old. However, for elderly patients with nonvalvular atrial fibrillation and Ccr (creatinine clearance rate) of 15–30 mL/min, a lower dose of 10 mg/d is advised.

Apixaban is a direct oral factor Xa inhibitor, similar to rivaroxaban, that regulates thrombin synthesis in anticoagulation for AF. It is superior to warfarin in preventing stroke, reducing bleeding events, and decreasing mortality in AF patients [39]. Apixaban is primarily metabolized by the liver and excreted through the digestive tract and kidneys, requiring caution in patients with liver injury. The standard dose is 5 mg twice daily, but for patients with renal insufficiency, a reduced dose of 2.5 mg twice daily is recommended based on the AVERROES and ARISTOTLE [40] trials, especially in elderly patients, those with low body weight, and those with high creatinine levels. These studies have shown similar efficacy and safety of apixaban compared to warfarin and aspirin in patients with normal renal function and even renal failure [41, 42, 43, 44]. However, limited data are available on the thrombolytic effect of apixaban and its comparison to VKAs, with small sample sizes and no statistically significant differences observed. Understanding the thrombolytic mechanism of apixaban highlights its advantage in shifting the coagulation/fibrinolytic balance toward fibrinolytic activity.

Edoxaban is a newly approved direct factor Xa inhibitor, similar to rivaroxaban and apixaban. One of the distinguishing features of edoxaban is that it does not interact with the cytochrome P-450 system. This makes edoxaban a viable option for patients with AF who require oral anticoagulation and have interactions with the cytochrome P-450 system. Regarding edoxaban dosing, Weitz *et al*. [45] conducted a phase II trial comparing various doses against warfarin; results showed that edoxaban at doses of 30/60 mg/d had similar efficacy and safety profiles. However, data regarding its efficacy in thrombolysis are limited. Only one study [21] directly compared edoxaban to warfarin in thrombolysis, but it did not provide further comparative data on the thrombolytic effect. More research is needed to investigate the comparative efficacy and safety of edoxaban and VKAs in thrombolysis.

NOACs have demonstrated significant advantages in treating left atrial thrombi, with differences in efficacy influenced by factors such as pharmacokinetics and drug targets. A meta-analysis study indicates that NOACs like rivaroxaban and dabigatran show promise in managing non-organized cardiac thrombi early on, potentially as standalone treatments without the need for additional anticoagulants [46]. Compared to VKAs, NOACs offer more predictable pharmacokinetics and pharmacodynamics, ensuring better stability in anticoagulation effects and reducing thrombosis or bleeding risks [47]. Moreover, NOACs exhibit limited drug interactions, providing convenience for patients without the risk of reduced efficacy or increased side effects due to interactions. With fixed dosages and reduced need for frequent laboratory monitoring, NOACs positively impact patient compliance and treatment outcomes [48]. The rapid absorption post-oral administration allows for quick onset of action, especially beneficial in acute situations for timely management of atrial thrombi [28]. The diverse efficacy among different NOACs can be explained by their pharmacokinetic variations, including differences in absorption, distribution, metabolism, and elimination, which directly influence their efficacy in anticoagulation. For instance, Rivaroxaban’s high bioavailability and moderate half-life enable effective coagulation control within a day [45]. NOACs acting on different coagulation factors, such as direct thrombin inhibitors (like Dabigatran) and direct Factor Xa inhibitors (like Rivaroxaban), have distinct mechanisms impacting their clinical outcomes [49]. Individual physiological differences like age, weight, and liver or kidney function can also influence NOAC efficacy, potentially leading to varied treatment responses among patients [50]. Furthermore, variations in recommended dosages and administration methods among NOACs can affect their clinical efficacy and safety.

This meta-analysis divided the included studies into subgroups based on the follow-up time and study type. The results showed that NOACs consistently demonstrated better thrombolysis effects than VKAs in different follow-up times and study types. Dabigatran and apixaban had higher absolute thrombus resolution rates, although significant differences were not observed, likely due to the small sample sizes. Comparisons between dabigatran and rivaroxaban showed similar thrombolytic rates [51]. However, larger clinical trials are needed for further confirmation. During the treatment process, some patients underwent a switch in thrombolytic regimens; this led to successful thrombus resolution. Increasing the dose of the original drug or switching to another NOAC regimen (such as between rivaroxaban and dabigatran) showed good thrombolytic effects [20, 23, 52]. However, careful monitoring for bleeding risk is crucial when increasing the dose of the original thrombolytic agent.

Patients with increased left ventricular end-diastolic inner diameter are at higher risk of developing left ventricular thrombosis due to abnormal ventricular wall movement and the presence of blood vortex flow in the ventricle. A previous cohort study has shown a low risk of LAT [30]. Factors such as an increase in LAD and decreased LVEF, along with persistent AF, are associated with a higher risk of thrombus formation in AF patients and can also affect thrombus resolution [18, 53]. However, current research on predicting LAT factors is limited. This meta-analysis compared the data from three studies [18, 20, 21] on thrombolysis effectiveness and ineffectiveness. The results indicated that patients with ineffective thrombolysis had a decrease in LVEDd and an increase in LAD, with statistically significant differences compared with those with effective thrombolysis. However, the analysis did not reflect the differences in AF subtype and LVEF between the effective and ineffective groups due to the limited number of included studies, small sample sizes, and potential bias. These findings suggest the need for further investigation into the impact of persistent AF, LVEDd, LAD, and LVEF on LAT. Improving these factors may potentially enhance thrombus resolution in patients with NVAF and LAT.

In this comprehensive meta-analysis evaluating the efficacy and safety of NOACs and VKA in thrombolysis for Atrial Fibrillation with LAT/LAAT, minimal heterogeneity and high homogeneity were observed. The heterogeneity of each outcome index remained stable, unaffected by subgroup analyses or iteratively excluding literature. Despite variations in subject demographics and comorbidities across studies, excluding individual studies showed no significant impact on the consistent and dependable results obtained. However, notable heterogeneity was identified in the comparison of LVEF index between effective and ineffective thrombolysis groups, with limited data available for subgroup analysis, posing challenges in assessing heterogeneity. In the Nelles *et al*. [21] study, the absence of Left Atrial Appendage closure and alterations in anticoagulant therapy due to unresolved atrial thrombus underscore the need for cautious interpretation of combined index analysis results.

All of the included studies in this analysis used the CHA2DS2-VASc scoring system to assess the stroke risk in patients with AF. The results indicated that the incidence of embolic events was 3.4% with NOACs compared with 7.7% with VKAs, although this difference was not statistically significant (*p* = 0.06). Similarly, the incidence of bleeding events during thrombolysis was 8.1% with NOACs and 9.9% with VKAs, and no statistically significant difference was observed (*p* = 0.77). Only two studies [21, 22] reported on mortality events, and the analysis showed no significant difference in mortality risk between NVAF patients with LAT and those treated with VKAs.

### Limitations of this Study

Numerous limitations in this study warrant acknowledgment. Firstly, the predominant inclusion of cohort studies over RCTs poses a limitation, given the inherent difficulties in achieving double-blinding and randomization with antithrombotic drugs. Secondly, while the efficacy indicators were thorough, the absence of safety indicators and other influencing factors on thrombus resolution in the literature may have introduced bias, potentially diminishing result credibility. As a solution, future research should prioritize large-scale multicenter RCTs to mitigate these challenges and strengthen the evidence base. Additionally, exploring concrete research avenues such as comparative dose studies for different NOACs could enhance the understanding of their efficacy in resolving atrial fibrillation with left atrial/left atrial appendage thrombus and provide further insights into optimal treatment strategies.

## 5. Conclusions

NOACs have a higher thrombolysis rate in patients with NVAF than VKAs. The use of NOACs does not significantly increase the risk of adverse events such as embolism, bleeding, and death compared with VKAs.

## Availability of Data and Materials

Data availability is not applicable to this article as no new data were created or analyzed in this study.

## Supplementary Material

Supplementary material associated with this article can be found, in the online version, at https://doi.org/10.31083/RCM26055.
